# Neuroprotective effects of cobalt oxide nanoparticles through mitigating oxidative stress and reactive glial responses in traumatic brain injury

**DOI:** 10.1016/j.mtbio.2025.102434

**Published:** 2025-10-16

**Authors:** Xuecheng Qiu, Congxin Shen, Yanyan Li, Mengwen Shao, Beibei Wang, Jingzhen Li, Jian-Feng Wei, Suning Ping, Wenshu Cong, Meng Li

**Affiliations:** a Jiangsu Key Laboratory of Brain Disease Bioinformation, Xuzhou Medical University, Xuzhou, Jiangsu, 221004, China; b Department of Biochemistry, School of Basic Medical Sciences, Xuzhou Medical University, Xuzhou, Jiangsu, 221004, China; c Department of Histology and Embryology, School of Basic Medical Sciences, Xuzhou Medical University, Xuzhou, Jiangsu, 221004, China; d Department of Histology and Embryology, School of Medicine, Shenzhen Campus of Sun Yat-Sen University, Shenzhen, Guangdong, 518107, China; e Neurobiology Research Center, School of Medicine, Shenzhen Campus of Sun Yat-Sen University, Shenzhen, Guangdong, 518107, China; f Beijing Center for Disease Prevention and Control, Beijing Key Laboratory of Diagnostic and Traceability Technologies for Food Poisoning, Beijing, 100013, China

**Keywords:** Traumatic brain injury, Nanoparticles, Cobalt oxide, Neuroprotection, Glia response, Oxidative stress

## Abstract

Traumatic brain injury (TBI) is a leading global cause of mortality and long-term neurological deficits. TBI involves primary mechanical damage and secondary injury processes, including oxidative stress and reactive glial responses. Oxidative stress, driven by excessive reactive oxygen species (ROS), exacerbates neuronal damage, causing cellular apoptosis and prolonged inflammation. Traditional antioxidants have limited efficacy due to poor bioavailability and targeting. Nanotechnology-based antioxidants offer a promising alternative due to their intrinsic antioxidative activities. In the present study, we explored the neuroprotective effects of dimercaptosuccinic acid-coated cobalt oxide nanoparticles (Co_3_O_4_ NPs) in a mouse TBI model. Co_3_O_4_ NPs significantly reduced oxidative stress, neuronal death, brain tissue loss, and reactive glial activation, thereby enhancing long-term motor function recovery. Mechanistically, Co_3_O_4_ NPs lowered neuronal ROS levels. Co_3_O_4_ NPs also decreased astrocytic NOX2 expression, mitigating reactive glial responses. These findings suggest that Co_3_O_4_ NPs might offer therapeutic potential for TBI.

## Graphical abstract

**Figure:**
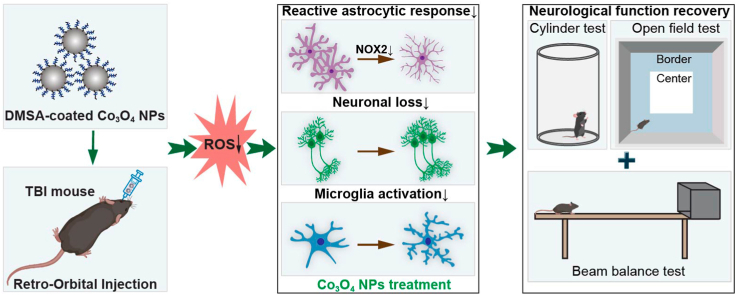

## 1 Introduction

Traumatic brain injury (TBI) is a significant global health concern, which has been a major cause of death and long-term neurological deficits worldwide [1,2]. TBI encompasses complex pathological changes, which include both primary and secondary injuries [3]. The primary injury involves direct mechanical damage to the brain, often resulting in a skull fracture and localized shearing and stretching damage at the core injury site, such as vascular disruption and axonal injury [3,4]. The primary injury initiates a series of molecular processes that promote the secondary injury processes, including excitotoxicity, microglial activation, reactive astrogliosis, lipid peroxidation, and oxidative stress, further accelerating neuronal death and neural degeneration [3,4].

Oxidative stress, primarily caused by excessive production of reactive oxygen species (ROS), has been recognized as a pivotal contributor to the secondary injury cascade following TBI [5,6]. ROS accumulation not only leads to cellular apoptosis and blood-brain barrier (BBB) disruption, but also promotes prolonged inflammatory response, thereby exacerbating secondary neuronal damage [7,8]. In recent years, the role of oxidative stress in TBI has drawn attention to the development of antioxidant therapies. Antioxidant therapies have facilitated functional recovery in the TBI model and provide a promising strategy for TBI patients [5,9]. Traditional antioxidants, such as ascorbic acid and N-acetyl-cysteine, have shown some efficacy in reducing oxidative damage but are often limited by poor bioavailability, short half-life, or insufficient targeting to the affected brain regions [10,11]. As a result, there is an increasing interest in developing more stable and effective antioxidants as therapeutics for TBI, such as nanotechnology-based antioxidants.

Nanoparticles, particularly those with antioxidative properties, have shown promise as neuroprotective agents in TBI treatment. Due to their small size, high surface area, and modifiable surface properties, nanoparticles can effectively penetrate the BBB and provide sustained ROS scavenging capabilities [12]. Cobalt oxide nanoparticles (Co_3_O_4_ NPs) have garnered significant attention due to their remarkable intrinsic catalase-like, peroxidase-like, and superoxide dismutase (SOD)-like activities [13]. Antimicrobial test and hemolysis assay have demonstrated that the synthesized Co_3_O_4_ NPs exhibit both efficient antioxidant and antibacterial properties [14]. Moreover, our previous study has revealed that dimercaptosuccinic acid (DMSA)-modified Co_3_O_4_ NPs alleviate aging and behavioral decline through the activation of the mitochondrial unfolded protein response (UPR) signaling pathway in *Caenorhabditis elegans* [15]. The above findings suggest the potential applicability of Co_3_O_4_ NPs in treating oxidative stress-associated diseases.

This study aims to assess the protective effects of Co_3_O_4_ NPs in TBI, particularly their capacity to reduce oxidative stress and ameliorate TBI-induced behavioral deficits. We hypothesize that Co_3_O_4_ NPs can mitigate oxidative damage, inhibit neuroinflammation and reactive astrogliosis, reduce neuronal loss, and thereby facilitate brain repair by lowering reactive oxygen species (ROS) levels. The present study may offer valuable insights into TBI treatment strategies and highlight the potential of Co_3_O_4_ NPs as an effective therapeutic intervention for oxidative stress-associated neurological diseases.

## 2 Materials and methods

### 2.1 Experimental animals

Male C57BL/6J mice (GemPharmatech, Jiangsu, China; 10–12 weeks) were kept under a 12-h light/dark cycle with *ad libitum* access to food and water. All experimental procedures in this study were approved by the Institutional Animal Care and Use Committee of Xuzhou Medical University.

### 2.2 Preparation and characterization of cobalt oxide nanoparticles

Cobalt oxide nanoparticles (Co_3_O_4_ NPs) were purchased from Aladdin (Shanghai, China). For the coating of dimercaptosuccinic acid (DMSA), Co_3_O_4_ NPs were incubated with 10 mg/mL DMSA solution (dissolved in ethanol) overnight at room temperature. After centrifugation, the Co_3_O_4_ NPs pellet was rinsed with ethanol three times and then dried at 50 °C. To measure the particle size and zeta potential of the Co_3_O_4_ NPs, the Co_3_O_4_ NPs were suspended with distilled water and then analyzed using Zetaview PMX-120 (Particle Metrix, Ammersee, Germany). To fluorescently label Co_3_O_4_ NPs with a fluorescent dye, Co_3_O_4_ NPs (10 mg/mL) were incubated with DiI (1,1′-dioctadecyl-3,3,3′,3′-tetramethylindocarbocyanine perchlorate) at a final concentration of 2 mg/mL for 4 h at room temperature with constant shaking. Then, the Co_3_O_4_ NPs were pelleted by centrifugation at 20,000 g for 10 min and rinsed with PBS three times with each rinse step lasting 1 h. The labeled Co_3_O_4_ NPs were resuspended in PBS.

### 2.3 Cytotoxicity analysis

Cell viability was evaluated using a CCK-8 Assay Kit (APExBIO, Boston, MA, USA) after incubating with various concentrations of Co_3_O_4_ NPs. HT22 or HEK293T cells were seeded in a 96-well plate with 100 μL cell medium overnight and then incubated with a range of concentrations of Co_3_O_4_ NPs for 24 h. Thereafter, 10 μL of CCK-8 working solution was added to each well, followed by an additional 1 h incubation. The absorbance at 450 nm was then determined using a microplate reader (BioTek Epoch2, Agilent Technologies, Santa Clara, CA, USA), and the relative cell viability was calculated.

### 2.4 ROS measurement

HT22 and HEK293T cells were seeded in a 12-well plate and then incubated with different concentrations of Co_3_O_4_ NPs for 16 h. Then, cells were incubated with DCFH-DA working solution (20 μM) at 37 °C for 30 min. After washing with DMEM medium, the cells were stimulated with H_2_O_2_ for 60–90 min and then washed with Dulbecco's phosphate-buffered saline (DPBS). The cells were collected, and fluorescence intensity was measured by BD FACSCanto™ II flow cytometry (BD biosciences, NJ, USA).

### 2.5 Annexin V and propidium iodide staining for apoptosis assay

To assess the role of Co_3_O_4_ NPs in H_2_O_2_-induced cell death, HT22 and HEK293T cells were seeded in a 12-well plate and then incubated with 10 μg/mL of Co_3_O_4_ NPs overnight. The next day, cells were treated with H_2_O_2_ (600 μM for HEK293T cells, 800 μM for HT22 cells) for 16 h. Cells were then harvested and stained with Annexin V-FITC/PI apoptosis detection kit (Vazyme, Wuhan, China) following the manufacturer's protocol. BD FACSCanto™ II flow cytometry (BD Biosciences, NJ, USA) was employed to detect cell apoptosis.

### 2.6 Traumatic brain injury model and treatments

A well-established weight-drop model of traumatic brain injury (TBI) was employed as previously described [16]. In brief, mice were administered an intraperitoneal injection of Avertin (Bidepharm, Shanghai, China) at a dosage of 250 mg/kg body weight for anesthesia. Subsequently, mice were secured on a stereotaxic frame. A 4-mm-diameter cranial window, centered 2 mm lateral to the Bregma, was created on the right side of the skull. The induction of TBI was achieved through the weight-drop method, where a steel block weighing 45 g, equipped with a 3-mm-diameter flat tip, was dropped from a height of 30 cm onto the exposed cortex, achieving an impact depth of 2 mm. The mice in the sham group were handled identically except for craniotomy and impact. After surgery, the mice were placed on a homoeothermic blanket set at 37 °C until they fully recovered from anesthesia. An analgesic (meloxicam, 10 mg/kg) was administered subcutaneously to relieve postoperative pain. For the treatment, Co_3_O_4_ NPs (at a dosage of 10 mg/kg body weight, dissolved in PBS) were administered to the mice via retro-orbital injection immediately following TBI, with the treatment lasting for three consecutive days. In the sham and vehicle groups, the mice received PBS injections as a control treatment.

### 2.7 Evaluation of the Co_3_O_4_ NPs biodistribution post treatment

To assess the biodistribution of the Co_3_O_4_ NPs, the DiI-labeled Co_3_O_4_ NPs (10 mg/kg body weight) were administered to the mice via retro-orbital injection immediately following TBI. For in vivo imaging, 6 h after DiI-labeled Co_3_O_4_ NPs injection, the mice were euthanized. Multiple organs, including the brain, heart, lung, liver, spleen, and kidney, were isolated and imaged using an IVIS Lumina S5 in vivo imaging system (PerkinElmer, Waltham, MA, USA). The biodistribution profile of the Co_3_O_4_ NPs was analyzed by the fluorescence intensity of DiI.

To identify which cell types uptook the Co_3_O_4_ NPs, DiI-labeled Co_3_O_4_ NPs were administered to the mice for three consecutive days. Six hours after the last administration, the mice were sacrificed and perfused with 4 % paraformaldehyde (PFA) solution, following the protocol detailed in the “Tissue Processing and Histological Immunostaining” section. The brain sections were co-immunostained with cell-type-specific markers, including neuron (NeuN), astrocyte (S100B), and microglia (IBA1), and imaged using a confocal microscope (Zeiss LSM 710, Carl Zeiss, Oberkochen, Germany) under Z-stack scan mode (with 1-μm interval) to identify the cell types that internalized the Co_3_O_4_ NPs.

### 2.8 Tissue processing and histological immunostaining

At the experiment's endpoint, the mice were euthanized by anesthetization with a lethal dose of Avertin. Subsequently, they underwent transcardial perfusion first with ice-cold 0.01 M phosphate-buffered saline (PBS) supplemented with 10 U/mL heparin and then with 4 % PFA solution. The mouse brains and other organs, including heart, lung, spleen, liver, and kidney, were isolated and post-fixed overnight at 4 °C in 4 % paraformaldehyde. Subsequently, they were dehydrated in a 30 % sucrose solution until they sank. Then, the brains were sectioned to a thickness of 20 μm using a cryostat (Leica Biosystems Inc., Wetzlar, Germany) and cryoprotected in an antifreeze solution (0.01 M PBS with 30 % ethylene glycol and 30 % glycerol, pH 7.2) at −30 °C until use. Other organs were sectioned at 10 μm thickness and directly mounted onto adhesive slides.

For free-floating immunofluorescence staining, two to three brain sections from Bregma +1 mm to −1 mm were selected and rinsed with PBS three times for 5 min each. The sections were then blocked with 10 % normal goat or donkey serum (depending on the host of the secondary antibodies) in 0.3 % Triton X-100 PBS for 1 h and followed by incubation with the primary antibody overnight at 4 °C. The primary antibodies were omitted in the negative control sections. The next day, brain sections were rinsed three times with PBS and incubated with corresponding secondary antibodies for 2 h at room temperature in the dark. After rinsing, brain sections were counterstained with 4’, 6-diamidino-2-phenylindole (DAPI) and mounted on the slides. The antibodies used in the present study are listed in Supplementary Table 1.

### 2.9 Quantification of immunofluorescence staining

Brain sections were imaged using a Zeiss confocal microscope (LSM710; Carl Zeiss, Oberkochen, Germany) under a 20 × or 40 × oil objective lens. Two image fields adjacent to the injury site (within 500 μm from the border of the lesion cavity) were taken in each brain section. To count the survival neurons, the number of NeuN-positive cells was automatically counted using the plugin “StarDist” in Fiji (ImageJ) software [17]. For the analysis of glial response following TBI, the GFAP-positive and IBA1-positive areas, as well as the integrated fluorescence intensity, were quantified using Fiji software with appropriate threshold adjustments. To analyze the morphological complexity of microglia, IBA1-positive cells containing nuclei (DAPI) were isolated and thresholded to binarized images. Subsequently, these binarized images were filtered based on particle size to eliminate noise and then skeletonized to enable Sholl analysis [18]. To analyze co-labeled images, the multi-channel image data were first separated into individual channels. Each channel of interest was converted to a binary format following the application of appropriate threshold adjustments. The colocalization area was acquired using the “Image Calculator” plugin in Fiji, with the “AND” logical operator and quantified using the “Measure” plugin integrated in Fiji.

### 2.10 Measurement of brain lesion volume

Serial sections from the whole brain with 200-μm intervals were collected and mounted onto adhesive slides (Vicmed, Jiangsu, China). Brain sections were stained with 0.1 % cresyl violet solution (Biosharp, Anhui, China) according to the manual. The brain sections were imaged using a stereomicroscope (Olympus SZX7, Tokyo, Japan). The total volume of brain lesions was calculated as: (total contralateral brain section area – total ipsilateral remaining brain section area) × section interval (200 μm).

### 2.11 Hematoxylin and Eosin staining

To explore the accumulation of Co_3_O_4_ NPs in various organs, the sections from the mouse brain, heart, lung, spleen, liver, and kidney were stained with a Hematoxylin and Eosin staining kit (Biosharp, Anhui, China) according to the manual instructions. Briefly, the sections were rinsed with water, immersed in the Hematoxylin solution for 5 min, and then rinsed with water three times. Subsequently, the sections were stained with Eosin solution for 10–30 s and rinsed with water. Then, the sections were dehydrated in a graded series of alcohols and cleared in xylene. Finally, the sections were mounted with mounting medium and imaged using a microscope (Olympus BX43, Tokyo, Japan).

### 2.12 Neurobehavioral tests

Behavioral tests were conducted in both subacute and chronic phases of TBI. Mice were transferred to a behavior test room and allowed to acclimate for 30 min before testing. The performance of the mice during the behavioral tests was monitored using the ANY-maze Video Tracking System (ANY-maze, IL, USA).

In the cylinder test, each mouse was placed into an acrylic cylinder (height: 20 cm; diameter: 10 cm), and their activity was recorded using a camera positioned at the bottom of the cylinder. A total of twenty rears were recorded within 10 min. Mice that failed the test were excluded. The forepaw contact with the cylinder wall and paw-dragging of the affected (contralateral) paw were assessed [19].

In the beam balance test, mice were trained to walk across a wooden strip (100 cm in length and 1 cm in width) three times to ensure successful passage. During the subacute and chronic phases of TBI, each mouse was placed on the wooden strip and allowed 2 min to traverse it, with three trials separated by intervals of more than 30 min. The time to cross the strip and the number of foot slips were recorded to evaluate the sensorimotor function of the mice.

In the open field test, each mouse was placed in the center of a 50 × 50 cm square arena and allowed to move freely for 10 min. Then, the total distance and immobile time were analyzed to assess the abnormal locomotor activity of each mouse after TBI.

In the Barnes maze test, mice underwent a three-day training regimen, each consisting of three trials, each lasting 2 min in a well-illuminated (about 1000 lux) testing room. The mouse was placed in the center of a Barnes maze apparatus (diameter of 120 cm, with 20 holes, one with an escape chamber). It was then permitted to explore until it entered the escape chamber or until a period of 2 min had elapsed. The mouse was manually placed into the escape chamber if it failed to find it. The mouse was allowed to stay in the escape chamber for 30 s during the training days. After three days of training, the mice were tested over an additional three days, with three trials conducted per day. The duration of finding the chamber in each trial was acquired. The following day, a probe test was conducted, and the moving distance within the target zone was analyzed to assess the spatial learning and memory abilities of the mice.

### 2.13 Total RNA extraction and transcriptome sequencing

Total RNA was isolated from the peri-injury regions at 6 h post the final treatment, following three consecutive days of treatment, using NcmSpin Cell/Tissue Total RNA Kit (NCM Biotech, Suzhou, China) in strict accordance with the manufacturer's recommended protocol. RNA quality was subsequently quantified using a NanoDrop ND-2000 spectrophotometer (NanoDrop Technologies, Wilmington, DE, USA) and further characterized for integrity using an Agilent 5300 Bioanalyzer (Agilent Technologies, Santa Clara, CA, USA). Only high-quality RNA samples (OD260/280 ≥ 1.95, RQN ≥6.5, 28S:18S ≥ 1.0) were used to construct sequencing library.

RNA library construction and high-throughput sequencing were conducted by Shanghai Majorbio Bio-pharm Biotechnology Co., Ltd. (Shanghai, China) in accordance with the standard operating protocols provided by the manufacturer. For the preparation of the RNA-seq transcriptome library, 1 μg of total RNA was used. The sequencing library was performed on the DNBSEQ-T7 platform (PE150) using DNBSEQ-T7RS Reagent Kit (FCL PE150) version 3.0 (Guangdong, China).

Raw paired-end reads underwent trimming and quality control using fastp [20] with default parameters. Subsequent to quality filtering, the resulting clean reads were individually aligned to the reference genome (i.e., Mus musculus genome assembly GRCm39) in orientation mode via the HISAT2 [21] alignment tool. For transcript assembly, the mapped reads from each sample were processed using StringTie [22], employing a reference-guided assembly strategy. The differential expression genes (DEGs) were identified using the DESeq2 [23]. DEGs with fold change ≥1.5 and q-value ≤0.05 were considered to be significantly different expressed genes. GO functional enrichment analysis was performed using the STRING online platform (https://cn.string-db.org/). The raw RNA sequencing data reported in this paper have been deposited in the Genome Sequence Archive (GSA) of the China National Center for Bioinformation (CNCB), under accession number CRA030153.

### 2.14 Statistical analysis

The data were presented as the mean ± standard error of the mean (SEM). The equality of variances was examined via the Brown–Forsythe test. When the sample size was adequate, the Shapiro–Wilk test was employed to assess the normality of distribution. For two-group comparisons, the student's t-test was applied to data with a normal distribution, whereas the Mann–Whitney test was utilized for data without a normal distribution. For multiple group comparisons, one-way analysis of variance (ANOVA), two-way ANOVA, or repeated two-way ANOVA was performed, followed by Tukey's HSD or Fisher's LSD test based on the data structure. Given the imbalance in sample sizes across experimental groups, a Welch's test was incorporated into the one/two-way ANOVA to account for violations of the homoscedasticity assumption inherent to conventional ANOVA. Two-tailed statistical significance tests were employed throughout the study, with a threshold of P < 0.05 indicating statistical significance.

## 3 Results

### 3.1 General characteristics and ROS scavenging activity of cobalt oxide nanoparticles

**Fig. 1:**
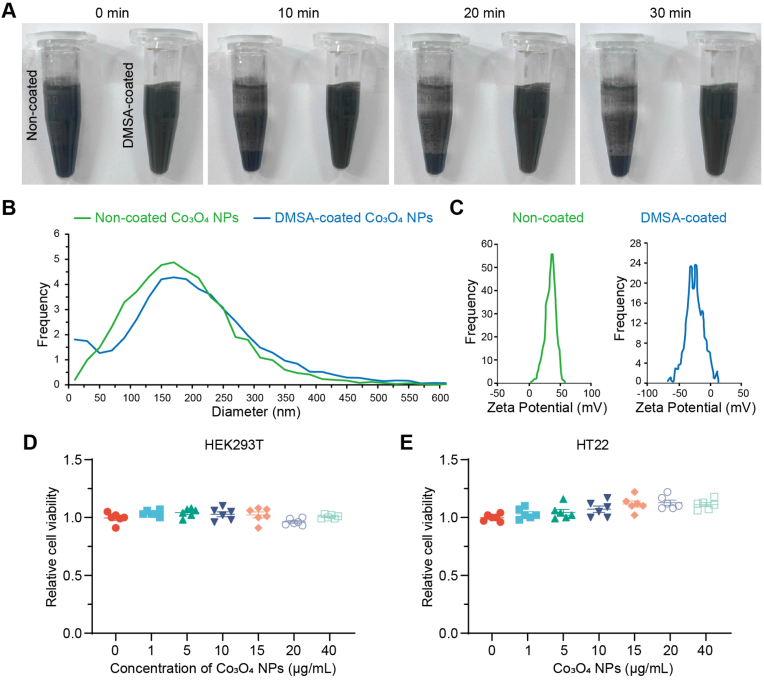
Characterization and biocompatibility of Co_3_O_4_ nanoparticles. (A) Water solubility and dispersibility of non-coated and DMSA-coated Co_3_O_4_ nanoparticles (NPs) over time. Co_3_O_4_ NPs were suspended in water, and sedimentation of Co_3_O_4_ NPs was observed at 0, 10, 20, and 30 min. (B) Size distributions of non-coated and DMSA-coated Co_3_O_4_ NPs. (C) Zeta potentials of non-coated and DMSA-coated Co_3_O_4_ NPs. (D, E) Biocompatibility of Co_3_O_4_ NPs. HEK293T (D) and mouse HT22 (E) cell lines were treated with various concentrations of Co_3_O_4_ NPs. The relative cell viability was measured at 24 h post-treatment by CCK-8 assay. Statistical analysis was performed using One-way ANOVA followed by Tukey's HSD test, n = 6.

To increase the stability of Co_3_O_4_ NPs in solution, we coated the Co_3_O_4_ NPs with DMSA to enhance the hydrophilicity. The DMSA-coated Co_3_O_4_ NPs exhibited markedly increased water solubility and dispersibility (Fig. 1A). Non-coated Co_3_O_4_ NPs rapidly sedimented within 10 min, whereas those coated with DMSA were stable as a suspension without notable sedimentation in water for at least 30 min (Fig. 1A). The size distribution of the DMSA-coated Co_3_O_4_ NPs was similar to our previous study (Fig. 1B). The zeta potential was smaller after coating with DMSA (Fig. 1C, non-coated: 34.56 ± 0.83 mV; DMSA-coated: 27.34 ± 1.69 mV), indicating successful deposition of the DMSA coating on the Co_3_O_4_ NPs [15]. Given their increased stability, DMSA-coated Co_3_O_4_ NPs, hereafter referred to as Co_3_O_4_ NPs, were used in the following experiments. Next, we assessed the biocompatibility of Co_3_O_4_ NPs using human HEK293T cells and mouse hippocampal neuronal cell line HT22 cells. No cytotoxicity was observed in either cell line at concentrations ranging from 1 to 40 μg/mL (Fig. 1D and E).

**Fig. 2:**
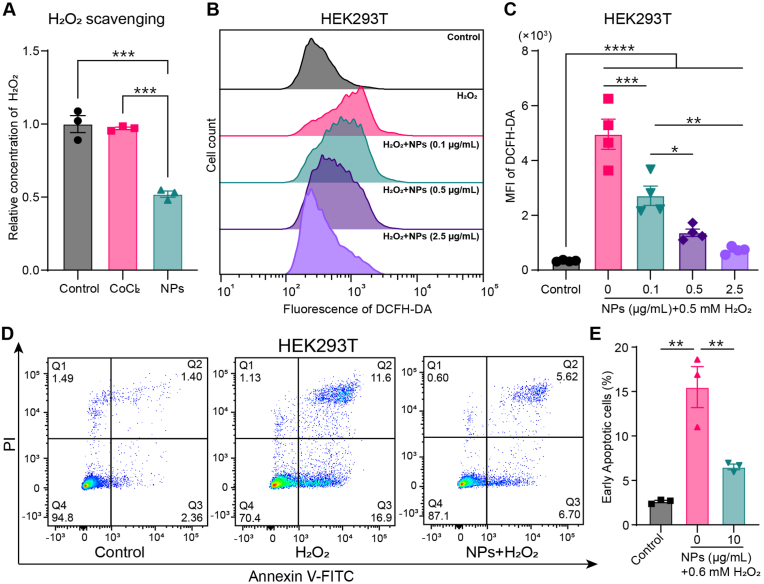
ROS scavenging activity of Co_3_O_4_ NPs in vitro. (A) Bar graph showing the H_2_O_2_ scavenging capacity of Co_3_O_4_ NPs in solutions (n = 3). (B) Representative histograms of ROS levels (DCFH-DA fluorescence) in HEK293T cells treated with H_2_O_2_ and various concentrations of Co_3_O_4_ NPs. (C) Quantification of mean fluorescence intensity (MFI) of DCFH-DA in HEK293T cells treated with H_2_O_2_ and various concentrations of Co_3_O_4_ NPs (n = 4). (D) Representative flow cytometry scatter plots of H_2_O_2_-induced early apoptosis in HEK293T cells after H_2_O_2_ and Co_3_O_4_ NPs treatments. Annexin V^+^/PI^−^ cells were quantified in (E) (n = 3). One-way ANOVA followed by Tukey's HSD test. *∗p* < 0.05, *∗∗p* < 0.01, *∗∗∗p* < 0.001, *∗∗∗∗p* < 0.0001.

It has been reported that Co_3_O_4_ NPs have intrinsic catalase-like and peroxidase-like activities [13]. Therefore, we assessed the capability of the Co_3_O_4_ NPs in scavenging H_2_O_2_ in solution and H_2_O_2_-induced ROS in cells. In H_2_O_2_ solution, approximately 50 % of total H_2_O_2_ was decomposed in the Co_3_O_4_ NPs group but not in the CoCl_2_ group (Fig. 2A). Pretreatment of HEK293T cells with Co_3_O_4_ NPs significantly decreased H_2_O_2_-induced ROS levels in the cells in a dose-dependent manner (Fig. 2B and C). Moreover, Co_3_O_4_ NPs reduced H_2_O_2_-induced early apoptosis of HEK293T cells (Fig. 2D and E). These results indicate that DMSA-coated Co_3_O_4_ NPs exhibit enhanced hydrophilicity and dispersibility, biocompatibility, and capability in scavenging reactive oxygen species.

### 3.2 Cobalt oxide nanoparticles accumulate in the damaged brain and exhibit favorable biocompatibility in vivo

**Fig. 3:**
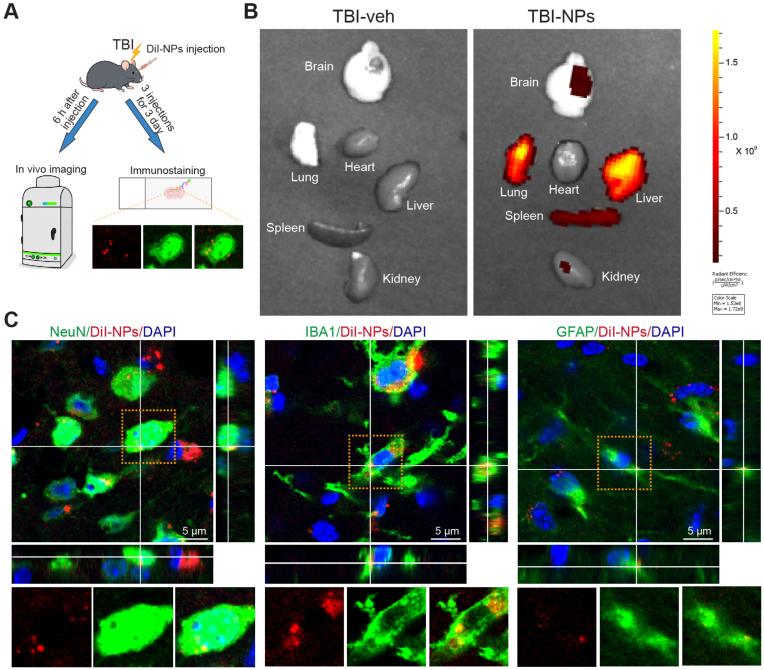
Biodistribution and cell uptaking of Co_3_O_4_ NPs after treatment in the acute phase of TBI. (A) Schematic flowchart illustrating the experimental design. (B) In vivo imaging demonstrating the in vivo distribution of DiI-labeled Co_3_O_4_ NPs in various organs, including the brain, heart, lung, liver, spleen, and kidney, after systematic administration. (C) Colocalization of DiI-labeled Co_3_O_4_ NPs with neurons (marked by NeuN), microglia (marked by IBA1), and astrocytes (marked by GFAP) in the brain, respectively. The result indicated that Co_3_O_4_ NPs can be internalized by various cell types of the central nervous system, including neurons, microglia, and astrocytes. Scale bar: 5 μm.

To evaluate the biodistribution and biocompatibility of Co_3_O_4_ NPs in the context of TBI, DiI-labeled Co_3_O_4_ NPs were administered immediately following TBI. At 6 h post-administration, the brains and peripheral organs (i.e., heart, liver, spleen, lungs, and kidneys) of the mice were harvested for in vivo imaging analysis. The DiI-labeled Co_3_O_4_ NPs exhibited predominant accumulation in the damaged regions of the brain, as well as in the lung, liver, and spleen (Fig. 3A and B). Within the perilesional region, Co_3_O_4_ NPs were internalized by neurons (marked by NeuN), microglia (marked by IBA1), and astrocytes (marked by GFAP) (Fig. 3C). The biocompatibility of Co_3_O_4_ NPs was assessed via hematoxylin-eosin (HE) staining. Histological examination demonstrated that Co_3_O_4_ NPs were detected in the injured brain (Supplementary Fig. 1A) and other organs, including the heart, liver, spleen, lungs, and kidneys (Supplementary Fig. 1B). No detectable impairments were observed in these organs following Co_3_O_4_ NPs treatment (Supplementary Fig. 1B). We further assessed the long-term biocompatibility of Co_3_O_4_ NPs at two additional doses (5 mg/kg and 20 mg/kg) at 1 month post-administration using biochemical assays for liver and kidney function. No statistically significant differences were observed when compared with the TBI-veh group (Supplementary Fig. 2A–J). Collectively, these findings demonstrate that Co_3_O_4_ NPs exhibit biocompatibility and accumulation in the damaged regions of the brain, which supports the therapeutic potential of Co_3_O_4_ NPs in preclinical TBI models.

### 3.3 Cobalt oxide nanoparticles treatment promotes neuron survival in the subacute phase of traumatic brain injury

**Fig. 4:**
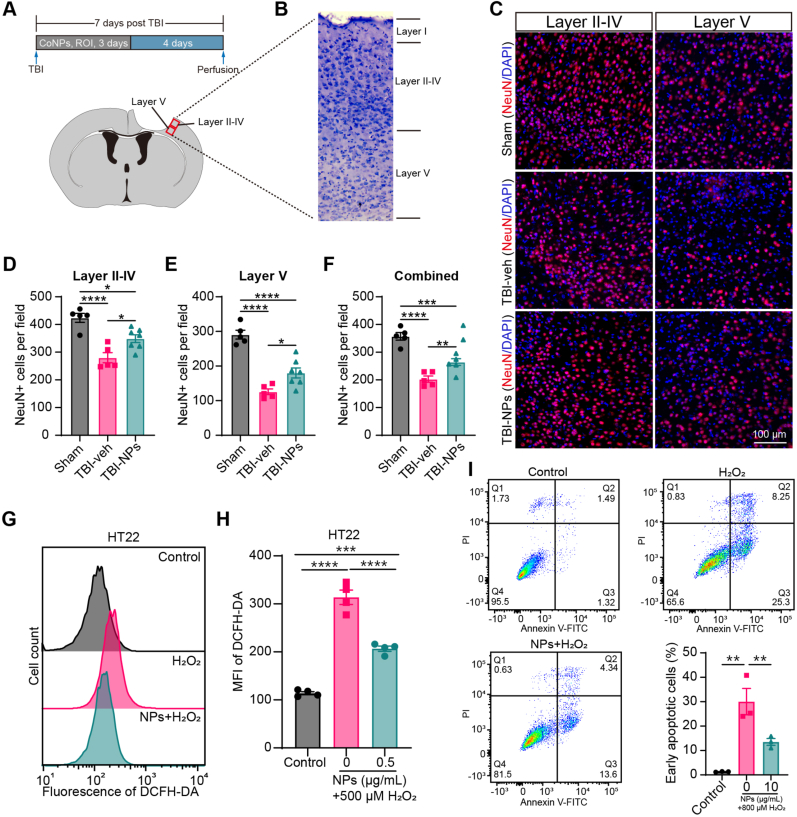
Treatment with Co_3_O_4_ NPs promotes neuron survival and prevents brain tissue loss post-TBI. (A) Schematic diagram of experimental design and imaging region. (B) Schematic illustration of different layers of the cortex for neuronal survival assessment. (C) Representative images of NeuN-positive neurons aside from the injury site in layers II-IV and V of the cortex. Scale bar: 100 μm. (D–F) Quantification of surviving neurons in different layers of the cortex (D: layer II-IV; E: layer V) as well as a combined result (F). One-way ANOVA followed by Tukey's HSD test. n = 5 in sham group, n = 5 in TBI-veh group, n = 7 in TBI-NPs group. (G) Representative histograms of ROS levels (DCFH-DA fluorescence) in mouse neuronal cells (HT22 cell line) after Co_3_O_4_ NPs treatment. (H) Quantification of ROS levels in HT22 cells. One-way ANOVA followed by Tukey's HSD test. n = 4. (I) Assessment of H_2_O_2_-induced early apoptosis of the HT22 cell line after Co_3_O_4_ NPs treatment using Live/Dead cell stain. One-way ANOVA followed by Tukey's HSD test. n = 3. *∗p* < 0.05, *∗∗p* < 0.01, *∗∗∗p* < 0.001, *∗∗∗∗p* < 0.0001.

Oxidative stress plays a crucial role in secondary injury following TBI and presents a potential therapeutic target for brain injury [6,10]. We analyzed single-cell RNA-sequencing datasets from the cortex of mice at the subacute phase of TBI (7 days post-TBI). We found significant upregulation of the ROS pathway in several cell types, including microglia, astrocytes, and neurons (Supplementary Fig. 3). To evaluate the neuroprotective role of Co_3_O_4_ NPs, mice subjected to TBI were treated with Co_3_O_4_ NPs for 3 days. Seven days after initial treatment, the neuronal survival adjacent to the injury site was assessed (Fig. 4A). Co_3_O_4_ NPs treatment significantly reduced TBI-induced neuronal loss in both layers II-IV and layer V of the injured cortex (Fig. 4B–F). In vitro results demonstrated that Co_3_O_4_ NPs treatment reduced H_2_O_2_-induced ROS production and prevented early apoptosis in the HT22 mouse hippocampal neuronal cells (Fig. 4G–I). These findings suggest that Co_3_O_4_ NPs treatment exerts a neuroprotective effect after TBI, likely by reducing neuronal oxidative damage.

### 3.4 Cobalt oxide nanoparticles treatment prevents reactive glial response after traumatic brain injury

**Fig. 5:**
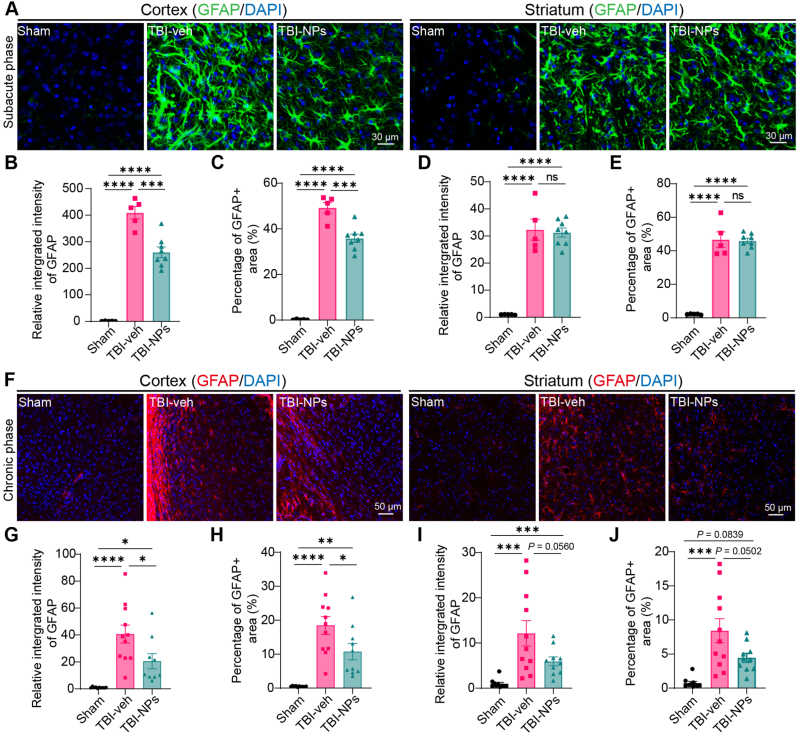
Co_3_O_4_ NPs treatment reduces reactive astrogliosis after TBI. (A) Representative images of GFAP immunostaining in the cortex and striatum of mice with different treatments in the subacute phase of TBI. Scale bar: 30 μm. (B, C) Quantification of the relative integrated intensity of GFAP-positive cells (B) and the percentage of GFAP-positive area (C) in the cortex of mice with different treatments during the subacute phase of TBI. (D, E) Quantification of the relative integrated intensity of GFAP-positive cells (D) and the percentage of GFAP-positive area (E) in the striatum during the subacute phase of TBI. Sample size in B–E: n = 5 in the sham group, n = 5 in the TBI-veh group, n = 8 in the TBI-NPs group. (F) Representative images of GFAP immunostaining in the cortex and striatum of mice in the chronic phase of TBI. Scale bar: 50 μm. (G, H) Quantification of the relative integrated intensity of GFAP-positive cells (G) and the percentage of GFAP-positive area (H) in the cortex during the chronic phase of TBI. (I, J) Quantification of the relative integrated intensity of GFAP-positive cells (I) and the percentage of GFAP-positive area (J) in the striatum during the chronic phase of TBI. One-way ANOVA followed by Tukey's HSD test. n = 10 in sham group, n = 11 in TBI-veh group, n = 10 in TBI-NPs group. *∗p* < 0.05, *∗∗p* < 0.01, *∗∗∗p* < 0.001, *∗∗∗∗p* < 0.0001.

Reactive glial response is a dynamic and multifaceted process that plays a crucial role in both the acute and chronic phases of TBI [24]. In this study, we examined the responses of astrocytes and microglia in TBI mice following Co_3_O_4_ NPs treatment. We explored the reactive astrocytic response during the subacute phase of TBI. Findings revealed that both the integrated intensity of GFAP fluorescence and the GFAP-positive area were significantly upregulated in the cortex and striatum (Fig. 5A–E). Treatment with Co_3_O_4_ NPs reduced the reactive astrocytic response in the cortex, yet had no such effect in the striatum (Fig. 5A–E). At three months post-TBI (chronic phase), treatment with Co_3_O_4_ NPs significantly reduced TBI-induced astrogliosis in both the cortex and striatum compared with the vehicle group (Fig. 5F–J). Overall, the above results suggest that treatment with Co_3_O_4_ NPs can alleviate reactive astrogliosis in both the subacute and chronic phases of TBI.

**Fig. 6:**
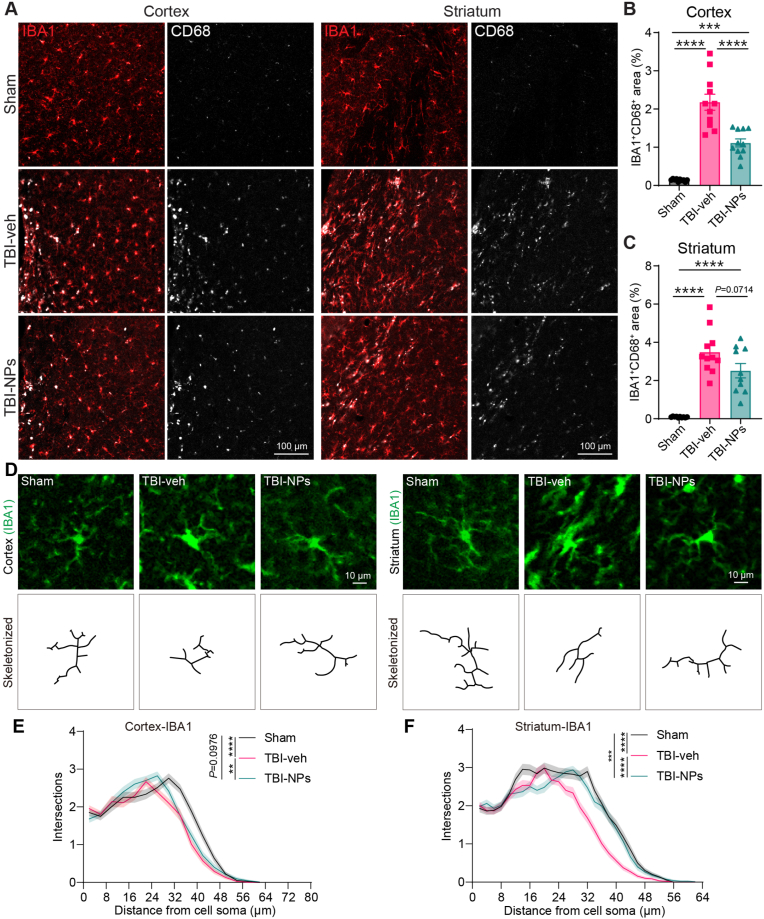
Co_3_O_4_ NPs treatment reduces microglial activation in the chronic phase of TBI. (A) Representative immunofluorescence images of IBA1 (a pan-microglial marker, red) and CD68 (a marker for activated microglia, gray) in the cortex and striatum of Sham, TBI-veh, and TBI-NPs groups. Scale bar: 100 μm. (B) Quantification of the percentage of IBA1^+^CD68^+^ area in the cortex across groups. (C) Quantification of the percentage of IBA1^+^CD68^+^ area in the striatum across groups. One-way ANOVA followed by Tukey's HSD test. n = 10 in sham group, n = 11 in TBI-veh group, n = 10 in TBI-NPs group. (D) Representative images of IBA1-labeled microglia and the skeletonized images in the cortex and striatum in the chronic phase of TBI. Scale bar: 10 μm. (E, F) Sholl analysis of individual microglial morphology in the cortex (E) and striatum (F) at 3 months post-TBI. Two-way ANOVA followed by Tukey's HSD test (main effect). Cortex: n = 78 in sham group, n = 84 in TBI-veh group, n = 80 in TBI-NPs group; striatum, n = 80 in sham group, n = 88 in TBI-veh group, n = 80 in TBI-NPs group. *∗p* < 0.05, *∗∗p* < 0.01, *∗∗∗p* < 0.001, *∗∗∗∗p* < 0.0001.

We investigated the potential role of Co_3_O_4_ NPs in microglial activation in the chronic phase of TBI (3 months post-TBI). The results showed that both the IBA1 positive area and the integrated intensity of IBA1 fluorescence in the cortex (Supplementary Fig. 4A–D) and striatum (Supplementary Fig. 4A-B, E-F) were increased in both the vehicle and Co_3_O_4_ NPs treatment groups after TBI. However, there were no significant differences between the vehicle and Co_3_O_4_ NPs treatment groups (Supplementary Fig. 4A–F). To further characterize the reactive microglial response, we performed co-immunostaining for IBA1 and CD68 (a marker of microglial activation) to assess microglial activation. TBI significantly upregulated activated microglia (IBA1^+^CD68^+^ double-positive) in both the cortex and striatum regions (Fig. 6A–C). Treatment with Co_3_O_4_ NPs resulted in a marked reduction of IBA1^+^CD68^+^ double-positive area in both the cortex (Fig. 6A and B) and striatum (Fig. 6A–C) compared to the TBI-veh group, indicating that Co_3_O_4_ NPs can reduce TBI-induced microglial activation.

The morphological changes in microglia serve as a sensitive marker for inflammation and severity of brain damage [25,26]. Resting microglia exhibit a ramified morphology, whereas reactive microglia typically display an amoeboid morphology [27]. We explored the complexity of microglia processes using the Sholl analysis following Co_3_O_4_ NPs treatment. The complexity of microglia processes was reduced after TBI (Fig. 6D–F). In contrast, treatment with Co_3_O_4_ NPs significantly increased the morphological complexity of microglia processes in both cortex (Fig. 6D and E) and striatum (Fig. 6D–F), indicating that Co_3_O_4_ NPs treatment could mitigate the reactive microglial response after TBI. Collectively, our findings suggest that Co_3_O_4_ NPs treatment ameliorates the TBI-induced reactive astrogliosis and microglial activation in both the cortex and striatum.

### 3.5 Cobalt oxide nanoparticles reduce astrocytic oxidative damage after traumatic brain injury

**Fig. 7:**
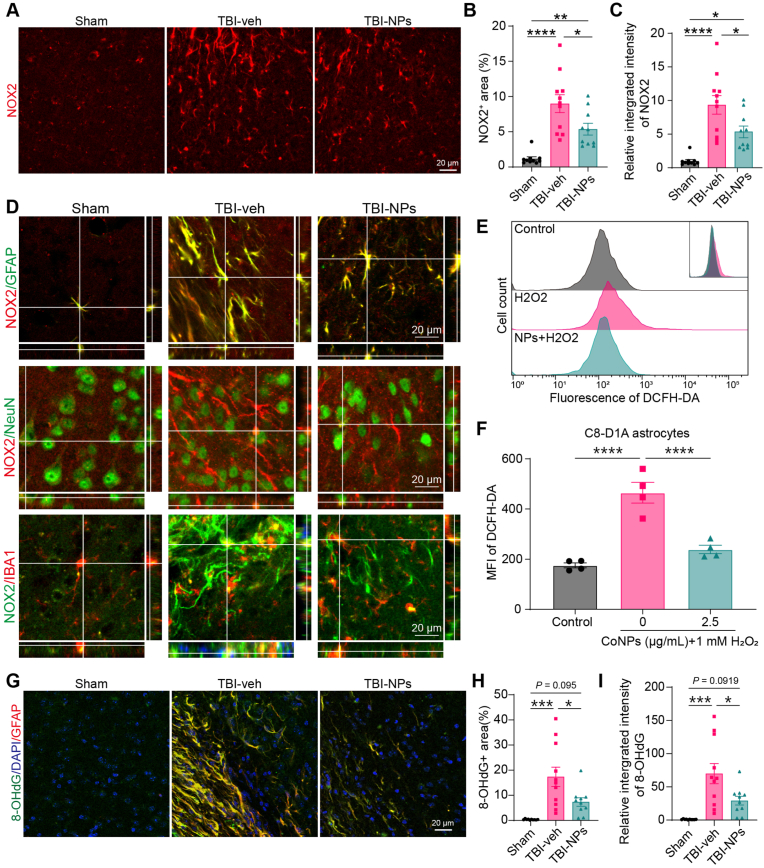
Treatment with Co_3_O_4_ NPs alleviates astrocytic oxidative injury after TBI. (A) Representative images of NOX2-positive immunostaining in the cortex of mice with different treatments. Scale bar: 20 μm. (B–C) Quantification of NOX2-positive area (B) and relative integrated intensity (C) of NOX2-positive immunostaining in the cortex of mice. One-way ANOVA followed by Tukey's HSD. n = 10 in the sham group, n = 11 in the TBI-veh group, n = 10 in the TBI-NPs group. (D) Co-immunostaining of NOX2 with astrocyte marker GFAP, neuron marker NeuN, and microglia marker IBA1 in the cortex at 3 months post-TBI. Scale bar, 20 μm. (E) Representative histogram of ROS levels in astrocytic cell line C8-D1A cells using DCFH-DA ROS probe. (H) Quantification of ROS levels in C8-D1A cells. One-way ANOVA followed by Tukey's HSD test. n = 3 for each group. (G) Double immunostaining showing GFAP co-stained with 8-OHdG (a biomarker of oxidative DNA damage) in the cortex of mice in different groups. (H, I) Quantification of 8-OHdG-positive area (H) and relative integrated intensity of 8-OHdG-positive staining (I) in the cortex of TBI mice. One-way ANOVA followed by Tukey's HSD test. n = 10 in the sham group, n = 11 in the TBI-veh group, n = 10 in the TBI-NPs group. *∗p* < 0.05, *∗∗p* < 0.01, *∗∗∗p* < 0.001, *∗∗∗∗p* < 0.0001.

NADPH oxidase (NOX) plays a pivotal role in the generation of ROS, which contributes to blood–brain barrier (BBB) disruption and neuronal death during brain injury [28,29]. NOX2 has been reported to induce oxidative stress, thereby promoting neuroinflammatory and neurotoxic responses following TBI [30]. Knockout of NOX2, rather than NOX4, promotes cell survival in TBI [31]. Our results demonstrated a significant increase in NOX2 expression during the chronic phase of TBI (3 months post-TBI, Fig. 7A–C). Treatment with Co_3_O_4_ NPs significantly reduced NOX2 expression (Fig. 7A–C). We then investigated the cell types with NOX2 upregulation following TBI. In the sham group, NOX2 was predominantly expressed in astrocytes (GFAP-positive cells) and minimally expressed in neurons (NeuN-positive cells) and microglia (IBA1-positive cells) (Fig. 7D). After TBI, the NOX2 was robustly upregulated in the astrocytes rather than neurons and microglia in both vehicle and Co_3_O_4_ NPs treatment groups (Fig. 7D). In vitro study demonstrated that treatment with Co_3_O_4_ NPs significantly decreased H_2_O_2_-induced ROS production in astrocyte cell line C8-D1A cell (Fig. 7E and F). Additionally, we employed 8-OHdG immunostaining to detect oxidative damage in DNA induced by ROS. Elevated levels of 8-OHdG are indicative of increased oxidative stress and DNA damage. Our results demonstrated that 8-OHdG immunostaining was predominantly observed in astrocytes (GFAP-positive cells) and significantly increased following TBI (Fig. 7G). Treatment with Co_3_O_4_ NPs reduced 8-OHdG-positive staining post-TBI (Fig. 5G–I). These findings suggest that Co_3_O_4_ NPs treatment could downregulate astrocytic NOX2 expression and mitigate oxidative damage after TBI.

### 3.6 RNA sequencing reveals that cobalt oxide nanoparticles regulate neurogenesis and oxidative stress-related gene expression in the acute phase of TBI

**Fig. 8:**
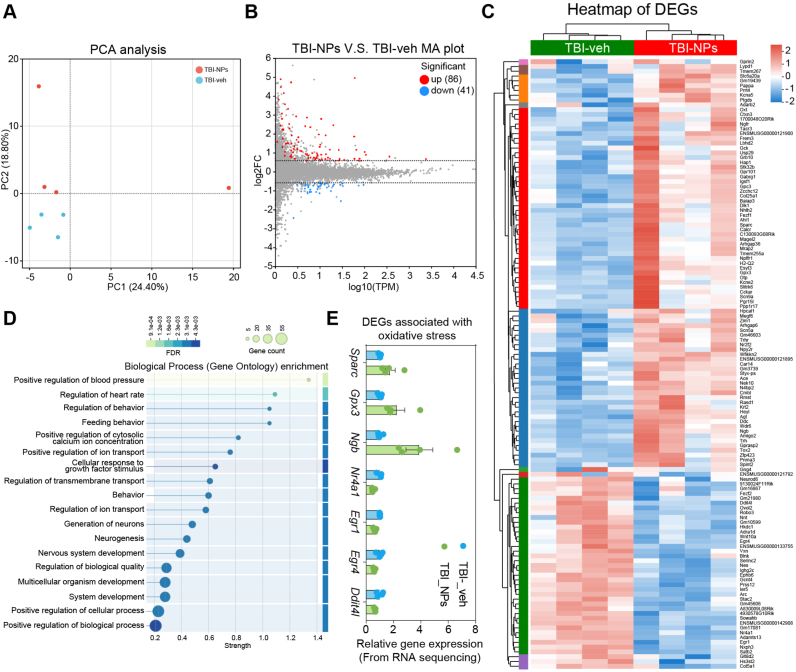
RNA sequencing reveals that Co_3_O_4_ NPs treatment regulates neural response and oxidative stress in the acute phase of TBI. (A) Principal Component Analysis (PCA) demonstrating a distinct separation between the TBI-NPs group and the TBI-veh group. (B) The MA plot showing the differential gene expression between the TBI-NPs and TBI-veh groups. There were 86 significantly up-regulated genes (red dots) and 41 significantly down-regulated genes (blue dots) in the TBI-NPs group compared to the TBI-veh group. (C) The heatmap of Differentially Expressed Genes (DEGs) between the TBI-veh and TBI-NPs groups. Genes with different expression levels were clustered. (D) Gene Ontology (GO) enrichment analysis for biological processes showing that DEGs were enriched in various processes. (E) Bar graph showing the DEGs (from RNA sequencing data) associated with oxidative stress.

To elucidate the therapeutic molecular mechanisms of Co_3_O_4_ NPs in TBI, we performed RNA sequencing (RNA-seq) after a three-day treatment with Co_3_O_4_ NPs. Principal Component analysis (PCA) showed a clear separation of gene expression profiles between the TBI-NPs and TBI-veh groups, indicating distinct transcriptional responses induced by Co_3_O_4_ NPs (Fig. 8A). The MA plot and heatmap of differentially expressed genes (DEGs) further revealed that Co_3_O_4_ NPs treatment led to significant changes in gene expression, with 86 genes upregulated and 41 genes downregulated (Fig. 8B and C). Gene Ontology (GO) enrichment analysis demonstrated that GO terms related to cellular response to growth factor stimulus, generation of neurons, neurogenesis, and nervous system development were enriched (Fig. 8D). Although oxidative stress-related GO terms were not significantly enriched, genes associated with anti-oxidative stress (e.g., *Sparc*, *Gpx3*, *Ngb*) were upregulated (Fig. 8E). Conversely, genes induced by oxidative stress (e.g., *Nr4a1*, *Egr1*, *Egr4*, *Ddit4l*) were downregulated (Fig. 8E), indicating that Co_3_O_4_ NPs may inhibit the cellular oxidative stress response. In summary, RNA-seq analysis elucidates that Co_3_O_4_ NPs exert regulatory effects on gene expression related to both neurogenesis and oxidative stress in the acute phase of TBI, providing molecular insights into their therapeutic potential.

### 3.7 Cobalt oxide nanoparticles treatment prevents chronic brain tissue loss and promotes neurological function recovery after traumatic brain injury

**Fig. 9:**
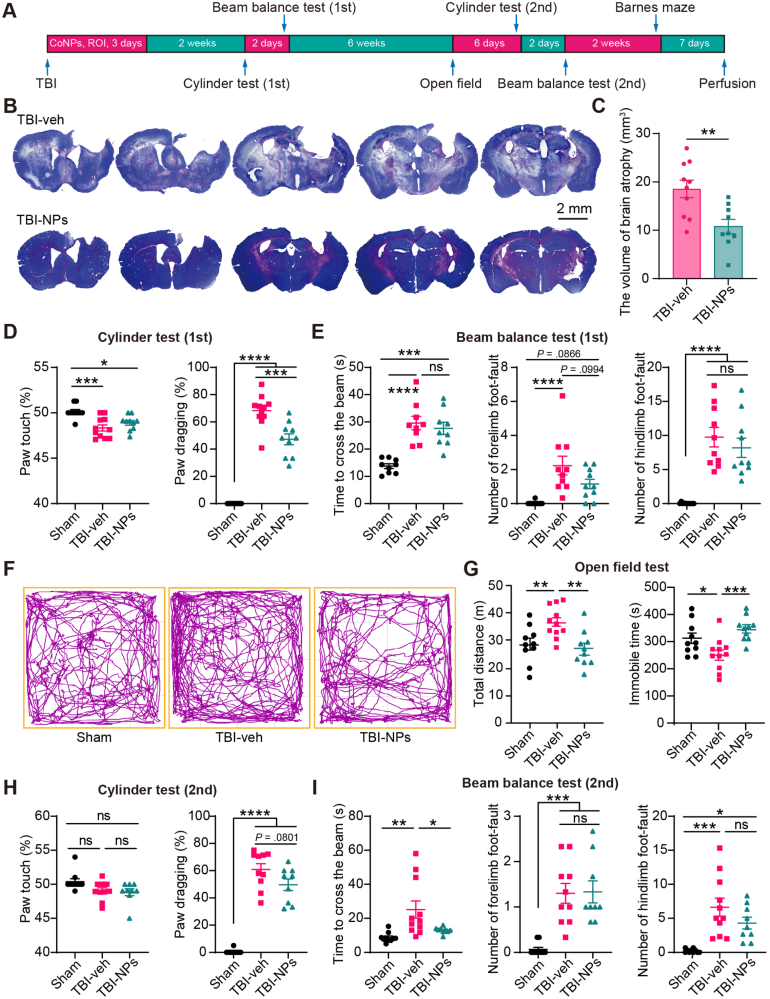
Co_3_O_4_ NPs treatment prevents chronic brain tissue loss and improves neurological function recovery in both subacute and chronic phases of TBI. (A) Flow chart of behavior tests and histological study. ROI, retro-orbital injection. (B) Representative Nissl staining of serial brain sections of the vehicle and Co_3_O_4_ NPs treated TBI mice. Scale bar: 2 mm. (C) Total volume of brain tissue loss after TBI in the vehicle and Co_3_O_4_ NPs treatment groups. Student's t-test. n = 10 in TBI-veh group, n = 9 in TBI-NPs group. (D) Quantification of the percentage of paw touches and paw dragging in the cylinder test in the subacute phase of TBI. (E) Quantification of the time to cross the beam and the number of forelimb and hindlimb foot-faults in the beam balance test in the subacute phase of TBI. (F) Representative traces of mice in the open field test in the chronic phase of TBI. (G) Quantification of the total distance and immobile time in the open field test. (H) Quantification of the percentage of paw touches and paw dragging in the cylinder test in the chronic phase of TBI. (I) Quantification of the time to cross the beam and the number of forelimb and hindlimb foot-faults in the beam balance test in the chronic phase of TBI. Original sample size: n = 10 in the sham group, n = 11 in the TBI-veh group, and n = 10 in the TBI-NPs group. Outliers were excluded using the ROUT method (Q = 1 %). One-way ANOVA followed by Tukey's HSD test. *∗p* < 0.05, *∗∗p* < 0.01, *∗∗∗p* < 0.001, *∗∗∗∗p* < 0.0001.

A single TBI insult can cause progressive and persistent neurological damage to patients, with limited therapeutic interventions showing long-term effects [32,33]. To evaluate the long-term benefits of Co_3_O_4_ NPs in TBI, we investigated brain atrophy and tissue loss at the chronic phase of TBI (Fig. 9A, post neurobehavioral tests). Our results demonstrated that Co_3_O_4_ NPs treatment significantly reduced brain tissue loss (Fig. 9B and C), indicating the long-term reparative effect of Co_3_O_4_ NPs treatment.

Given that the sensorimotor cortex was injured in our model, a series of neurobehavioral tests, including the cylinder test, beam balance test, open field test, and Barnes maze test, were conducted to evaluate the neurorestorative efficacy of Co_3_O_4_ NPs treatment during both the subacute and chronic phases of TBI (Fig. 9A). In the subacute phase of TBI, the cylinder test results showed a significant reduction in the percentage of paw touches and a significant increase in the percentage of paw dragging for the affected paw in the TBI group compared with the sham group (Fig. 9D), indicating that TBI significantly impaired sensorimotor function. Notably, treatment with Co_3_O_4_ NPs partially restored TBI-induced sensorimotor function impairment (Fig. 9D). Beam balance test showed mild but not significant improvement by Co_3_O_4_ NPs treatment (Fig. 9E). We also explored the dose-dependent therapeutic effects of Co3O4 NPs. Compared with the TBI-veh group, treated with both low (5 mg/kg) and high (20 mg/kg) doses of Co_3_O_4_ NPs significantly decreased paw dragging behavior in the cylinder test (Supplementary Fig. 5A). In the beam balance test, Treatment with the high dose (20 mg/kg) of Co_3_O_4_ NPs significantly reduced foot-faults of the hindlimb, whereas the low dose (5 mg/kg) had no effect (Supplementary Fig. 5B), indicating that Co_3_O_4_ NPs exert slightly dose-dependent therapeutic effects in TBI-induced motor impairment. These dose-dependent effects were validated through the integration of combined experimental results with different dose of Co_3_O_4_ NPs (5 mg/kg, 10 mg/kg, 20 mg/kg, Supplementary Fig. 5C–E). These findings suggest that Co_3_O_4_ NPs treatment slightly improves the motor balance and coordination in mice and exhibits a dose-dependent effect at the subacute phase of TBI.

In the chronic phase of TBI, we initially assessed motor activity in mice using the open field test. The TBI mice exhibited increased total moving distance and decreased immobile time (Fig. 9F and G), indicating a hyperactivity phenotype post-TBI [34,35]. Notably, treatment with Co_3_O_4_ NPs restored this abnormal motor activity (Fig. 9F and G). We re-evaluated the sensorimotor function and motor coordination in the chronic phase of TBI using the cylinder test and beam balance test, respectively. In the cylinder test, although the percentage of paw touch had no differences among sham, TBI-veh, and TBI-NPs groups, the percentage of paw dragging remained higher in the TBI-veh group (Fig. 9H). Treatment with Co_3_O_4_ NPs slightly reduced paw dragging post-TBI (Fig. 9H). In the beam balance test, Co_3_O_4_ NPs treatment significantly reduced the time required to pass through the beam, but not the foot-faults of the affected forelimb and hindlimb (Fig. 9I). We also explored the changes in spatial learning and memory function after treatment in TBI mice. The spatial learning and memory functions were impaired in mice with TBI. However, administration of Co_3_O_4_ NPs did not result in any discernible improvement (Supplementary Fig. 6A–C). Consistent with the histological observations, Co_3_O_4_ NPs were undetectable within the hippocampal CA1 region and their administration neither attenuated neuronal loss nor modulated aberrant synaptogenesis in the chronic phase of TBI (Supplementary Fig. 7A–D) [36]. Overall, our results suggest that Co_3_O_4_ NPs have a long-term therapeutic effect on improving sensorimotor function and motor coordination rather than the spatial learning and memory function post-TBI.

## 4 Discussion

Traumatic brain injury (TBI) can result in long-term neurodegeneration and structural disruptions in the brain, potentially causing permanent neural function impairments [37,38]. The pathophysiology of TBI involves complex primary and secondary injury processes. Oxidative stress has been a critical factor contributing to the secondary injury, including neuronal damage and dysfunction [6]. Recent research has increasingly focused on therapeutic strategies aimed at mitigating these secondary injury processes, particularly through the application of nanotechnology-based antioxidants [[39], [40], [41], [42]]. In this study, we explored the neuroprotective potential of cobalt oxide nanoparticles (Co_3_O_4_ NPs), which possess intrinsic antioxidant properties, as a treatment for oxidative stress and neuronal damage associated with TBI. Our results have demonstrated that treatment with Co_3_O_4_ NPs promotes neuronal survival, reduces brain tissue loss and reactive glial responses, and improves neurological function recovery in both the subacute and chronic phases of TBI. Mechanically, Co_3_O_4_ NPs promote the clearance of reactive oxygen species (ROS) and mitigate oxidative damage in neurons and glial cells after TBI.

The physicochemical properties of nanoparticles, including their small size and high surface area, offer a beneficial platform for biomedical applications, such as the targeted delivery of bioactive agents through surface modification of nanoparticles [43,44]. The modification of Co_3_O_4_ NPs with DMSA enhanced their hydrophilicity, thereby improving their stability and dispersion in aqueous solutions, which is crucial for biomedical applications [45]. Our previous study has revealed the anti-aging effects of Co_3_O_4_ NPs [15]. In the present study, we observed that Co_3_O_4_ NPs enhanced the ROS clearance in an H_2_O_2_-induced oxidative stress cell model, likely due to the peroxidase-like activity of Co_3_O_4_ NPs [13].

Following the TBI, a cascade of molecular events triggers inflammation, oxidative stress, and neuronal death [46]. In the present study, we observed that Co_3_O_4_ NPs were enriched in the damaged brain and internalized by neurons, microglia, and astrocytes, which prevented TBI-induced neuron loss and reactive glial response. Mechanistically, Co_3_O_4_ NPs can directly alleviate neuronal oxidative damage. In addition, a decreased reactive glial response can also reduce neurotoxicity, indirectly promoting neuronal survival [47]. Notably, TBI is increasingly recognized as a chronic condition, and the pathological progression can persist over several years, leading to progressive neurodegeneration and permanent neurological function deficits [38,[48], [49], [50]]. Our study demonstrated that Co_3_O_4_ NPs treatment improved sensorimotor function and motor coordination post-TBI not only in the subacute phase of TBI but also in the chronic phase of TBI. These behavioral improvements are consistent with our histological findings, which showed that Co_3_O_4_ NPs treatment significantly rescued TBI-induced neuronal loss in the injured cortex and reduced brain atrophy in the chronic phase of TBI. However, Co_3_O_4_ NPs treatment did not improve TBI-induced spatial learning and memory impairment. The spatial learning and memory impairment following TBI is severity-dependent [51]. The limited hippocampal impairment may prevent Co_3_O_4_ NPs from entering the hippocampus and exerting an effect, which was supported by the absence of DiI-labeled Co_3_O_4_ NPs in the CA1 subregion of the hippocampus and their minimal impact on TBI-induced neuronal loss and aberrant synaptogenesis in this area. Additionally, it is noteworthy that distinct improvements in behavioral indicators were observed within the same behavioral tests within the subacute and chronic phases of TBI, indicating the complexity of the pathological progression and the subsequent repair processes following brain injury.

The reactive glial response, characterized by astrogliosis and microglial activation, plays a crucial role in TBI pathology [4,52]. After TBI, astrocytes and microglia become reactive and undergo significant morphological changes [25,52]. While this activation initially serves as a protective mechanism to limit the extent of damage, it can also exacerbate neurodegeneration during the chronic phase of TBI [24,[52], [53], [54]]. Treatment with Co_3_O_4_ NPs significantly reduced astrocytic activation, as indicated by a decrease in the GFAP-positive area in both the cortex and striatum during the chronic stage of TBI. It is worth noting that Co_3_O_4_ NPs treatment had a limited role in microgliosis based on the IBA1-positive area. However, treatment with Co_3_O_4_ NPs reduced IBA1^+^CD68^+^ area and increased the morphological complexity of microglial processes, indicating a shift in the microglial response toward a less activated state.

Oxidative stress is one of the key mediators of secondary injury in TBI pathology [55], and NADPH oxidase 2 (NOX2) plays a major role in TBI-induced oxidative stress [56]. Inhibition of NOX2 can enhance neuroprotection and reduce neuroinflammation following TBI [56,57]. In the present study, we observed that NOX2 is primarily expressed in astrocytes. Treatment with Co_3_O_4_ NPs significantly reduced NOX2 expression in the chronic phase of TBI, suggesting that Co_3_O_4_ NPs can mitigate astrocytic oxidative damage. These findings are consistent with in vitro results showing that Co_3_O_4_ nanoparticles reduced H_2_O_2_-induced ROS production, as well as in vivo studies demonstrating that Co_3_O_4_ NPs treatment decreased astrocytic DNA damage (8-OHdG immunostaining). Overall, our results suggest that Co_3_O_4_ NPs may primarily target astrocytes, which subsequently influence microglial response indirectly, and further contribute to neuroprotection in the chronic phase of TBI [58,59].

TBI-induced oxidative stress is not limited only to the brain. It can also affect multiple organs, leading to widespread cellular damage [60,61]. Systemic antioxidant treatments have been shown to improve cognitive impairment and prevent the progression of neurodegenerative disorders by reducing oxidative stress throughout the body [[62], [63], [64]]. In the present study, we observed that Co_3_O_4_ NPs are deposited in damaged brain and multiple organs, which may enhance the local and systemic ROS clearance and mitigate inflammation after TBI. However, the molecular mechanisms by which Co_3_O_4_ NPs reduce oxidative stress and reactive glial response, especially in the acute phase of TBI, still remain incompletely understood. While our RNA sequencing (RNA-seq) analysis has identified the potential regulatory role of Co_3_O_4_ NPs in neurogenesis and oxidative stress (likely mediated via antioxidant-related genes), additional studies are necessary to establish more robust molecular evidence supporting these observed effects.

## 5 Conclusions

This study demonstrates that DMSA-coated Co_3_O_4_ nanoparticles (Co_3_O_4_ NPs) exhibit significant neuroprotective properties by reducing oxidative stress, neuronal apoptosis, and glial activation after TBI. Notably, Co_3_O_4_ NPs treatment significantly enhances neurological function recovery during both the subacute and chronic phases of TBI. Mechanistically, Co_3_O_4_ NPs upregulate anti-oxidative stress-associated gene expression and reduce ROS levels in both neurons and glial cells, thereby mitigating neuronal loss and reactive glial responses. Future studies should explore the potential modification of Co_3_O_4_ NPs to enhance therapeutic targeting and efficacy. Overall, our findings demonstrate the therapeutic potential of Co_3_O_4_ NPs in TBI and potentially in other neurodegenerative disorders.

## CRediT authorship contribution statement

**Xuecheng Qiu:** Writing – review & editing, Writing – original draft, Supervision, Project administration, Investigation, Funding acquisition, Conceptualization. **Congxin Shen:** Visualization, Methodology, Formal analysis, Data curation. **Yanyan Li:** Data curation. **Mengwen Shao:** Methodology. **Beibei Wang:** Data curation. **Jingzhen Li:** Writing – review & editing. **Jian-Feng Wei:** Writing – review & editing. **Suning Ping:** Writing – review & editing, Project administration, Conceptualization. **Wenshu Cong:** Writing – review & editing, Supervision, Conceptualization. **Meng Li:** Writing – review & editing, Supervision, Funding acquisition, Conceptualization.

## Declaration of competing interest

The authors declare no conflict of interest.

## Ethics approval statement

All animal experiment procedures were approved by the Institutional Animal Care and Use Committee of Xuzhou Medical University and performed following the National Institutes of Health Guide for the Care and Use of Laboratory Animals.

## Declaration of competing interest

The authors declare that they have no known competing financial interests or personal relationships that could have appeared to influence the work reported in this manuscript.

## Data Availability

The data used or analyzed during the current study are available from the corresponding author upon reasonable request. The raw RNA sequencing data reported in this paper have been deposited in the Genome Sequence Archive (GSA) of the China National Center for Bioinformation (CNCB), under accession number CRA030153.
