# Supplementary material for: Neuroprotective effects of cobalt oxide nanoparticles through mitigating oxidative stress and reactive glial responses in traumatic brain injury
Source: Mater Today Bio. 2025 Oct 16;35:102434. doi: 10.1016/j.mtbio.2025.102434 (PMC12593661; doi:10.1016/j.mtbio.2025.102434)
Supplement: Multimedia component 1 [file mmc1.docx]

**Neuroprotective effects of cobalt oxide nanoparticles through mitigating oxidative stress and reactive glial responses in traumatic brain injury**

Xuecheng Qiu, Congxin Shen, Yanyan Li, Mengwen Shao, Beibei Wang, Jingzhen Li, Jian-Feng Wei, Suning Ping, Wenshu Cong, Meng Li

**Supplementary Figure 1**


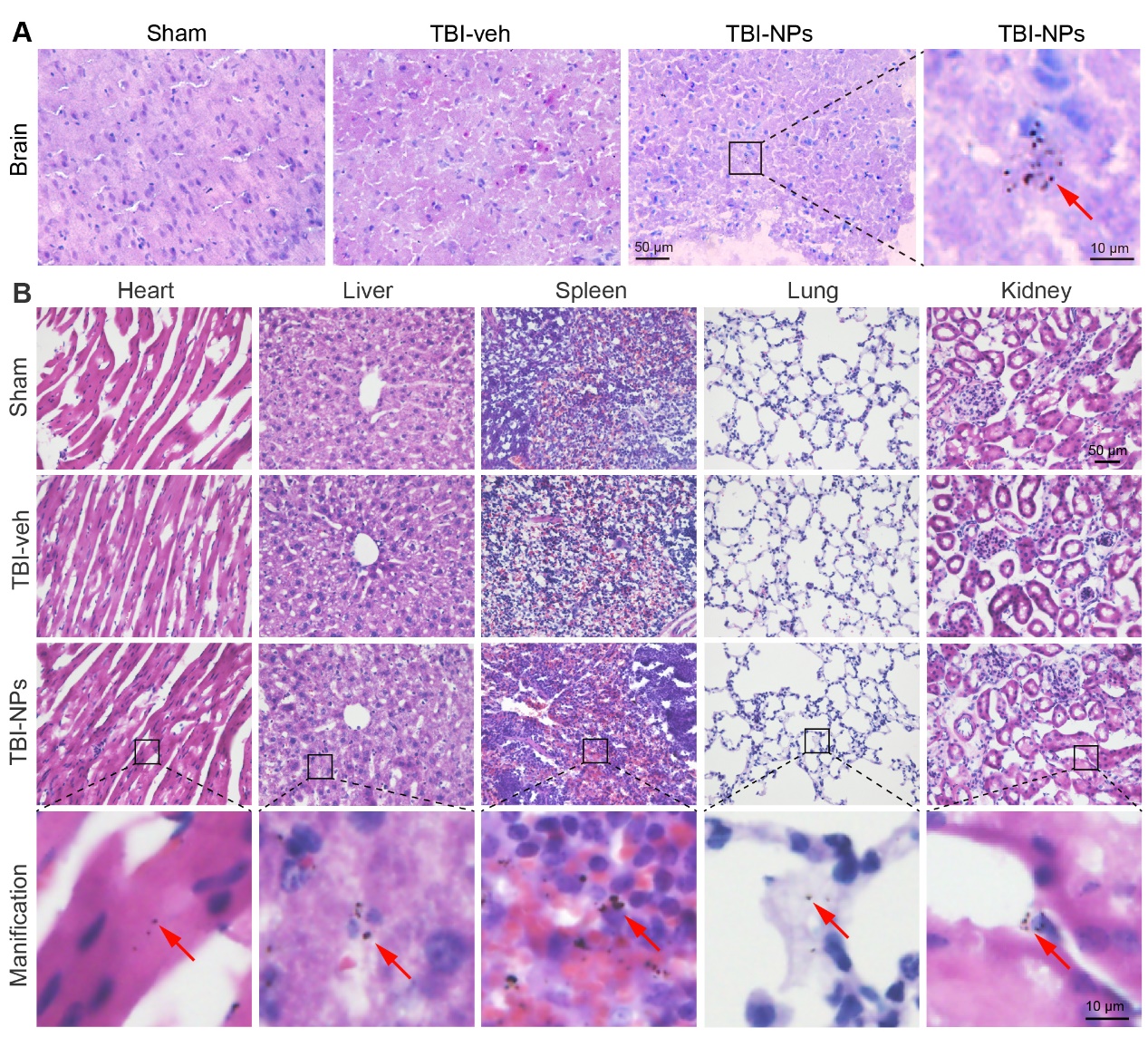


**Supplementary Figure 1.** Biocompatibility assessment of Co_3_O_4_ NPs treatment in vivo. (A-B) Representative hematoxylin and eosin (H&E) staining images showing Co_3_O_4_ NPs in the brain (A) and various organs (B) in Co_3_O_4_ NPs treated mice. Scale bar: 50 µm. Black boxes show fields of magnification (Scale bar: 10 µm). Red arrows indicate Co_3_O_4_ NPs deposited in organs.

**Supplementary Figure 2**


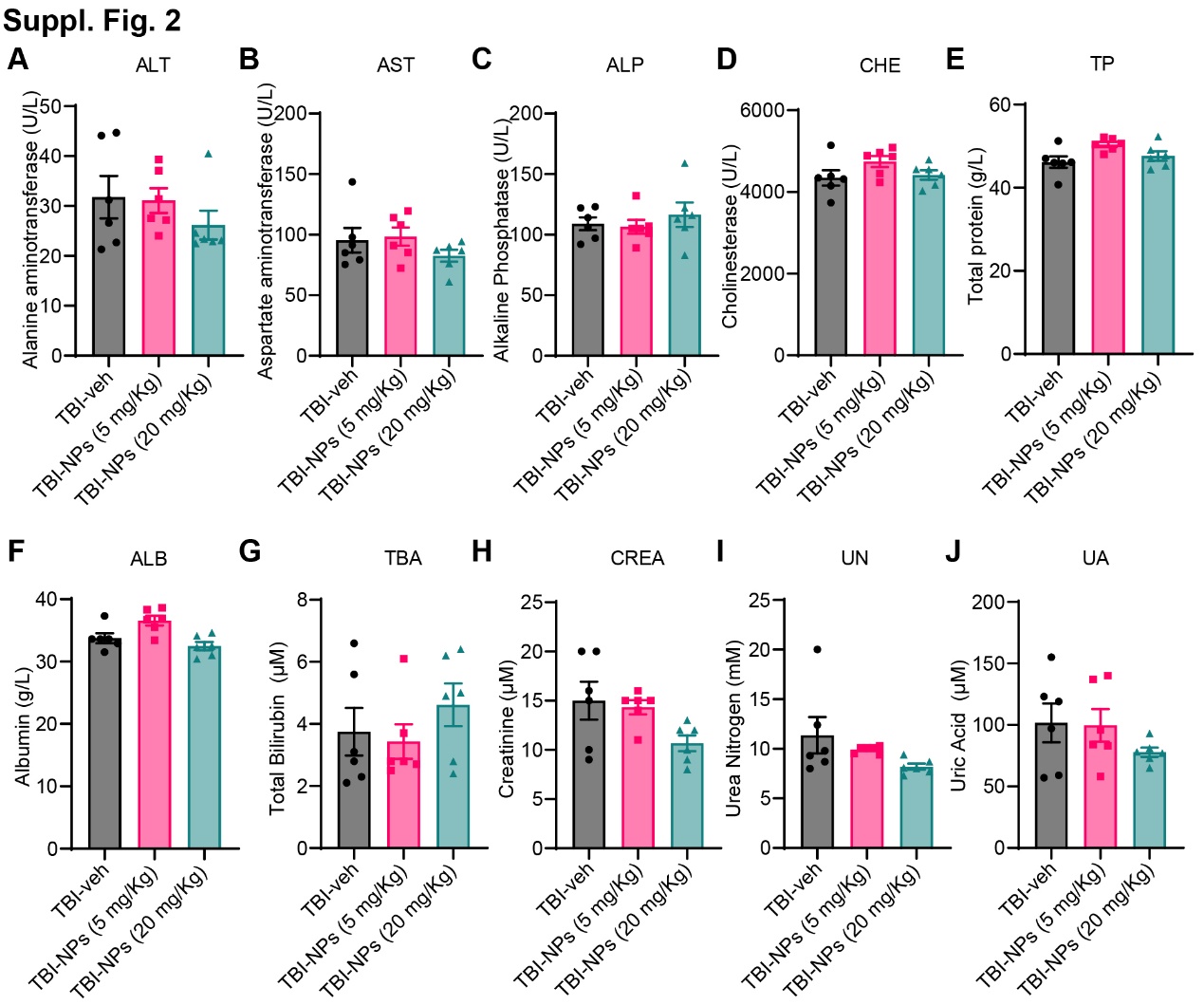


**Supplementary Figure 2.** Co_3_O_4_ NPs exhibit long-term biocompatibility in vivo. (A-J) The bar graphs illustrate the levels of various biochemical markers in the serum of mice treated with different concentrations of Co_3_O_4_ NPs (5 mg/kg and 20 mg/kg) compared to the TBI-veh group using the cobas® 8000 modular analyzer series (Roche). The markers assessed include liver function enzymes such as alanine aminotransferase (ALT, A), aspartate aminotransferase (AST, B), alkaline phosphatase (ALP, C), and cholinesterase (CHE, D), as well as total protein (TP, E), albumin (ALB, F), and total bilirubin (TBA, G). Kidney function was evaluated through the measurement of creatinine (CREA, H), urea nitrogen (UN, I), and uric acid (UA, J). Each group consists of n=6 mice. The results suggest that Co_3_O_4_ NPs at the tested concentrations do not significantly alter the levels of these biochemical markers, indicating no adverse effects on liver and kidney functions over the observed period. One-way ANOVA tests were performed.

**Supplementary Figure 3**


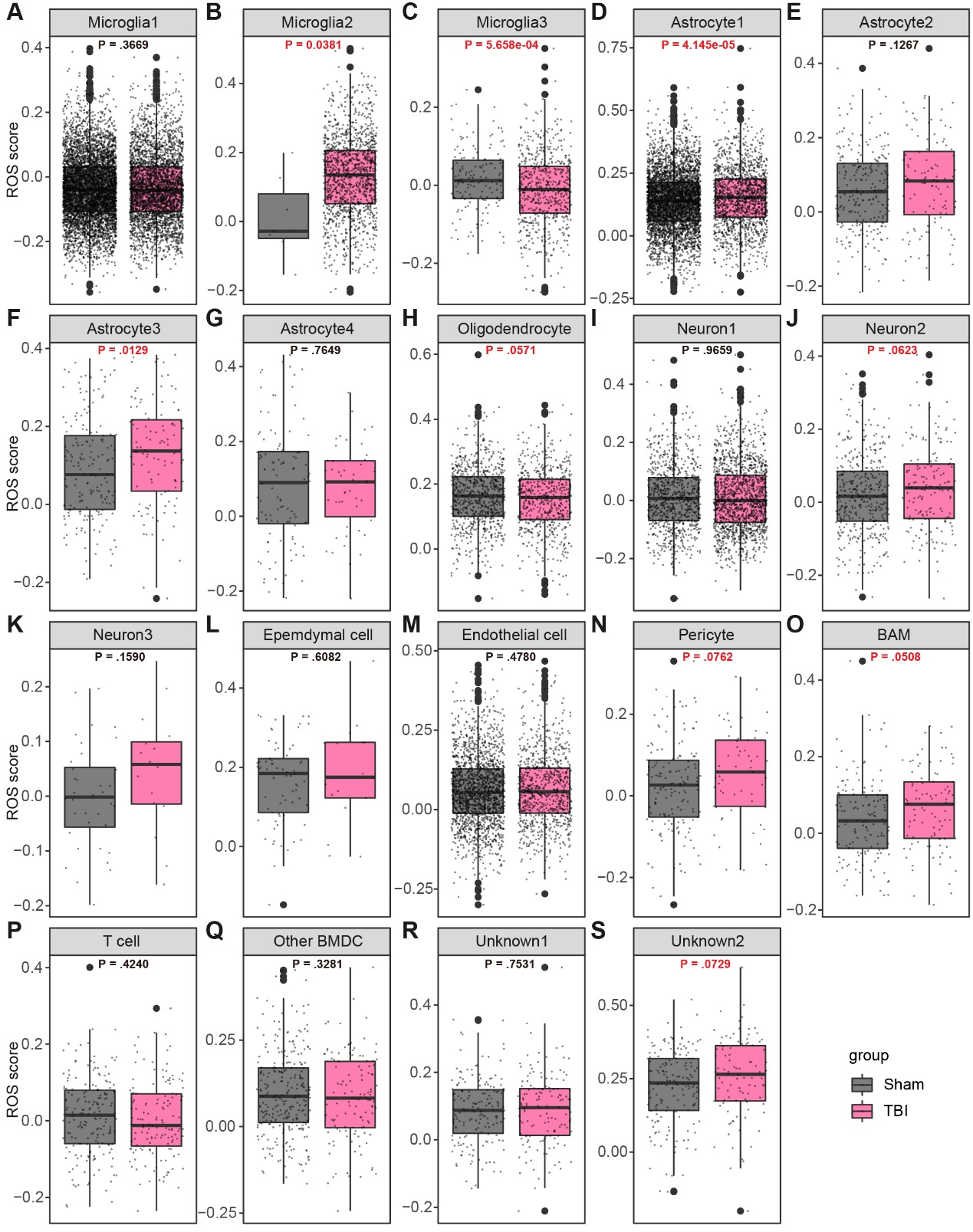


**Supplementary Figure 3.** Single-cell RNA-sequencing analysis demonstrates the ROS score of individual cell types at 7 days post-TBI. The single-cell RNA-sequencing data were collected from Gene Expression Omnibus (GEO, accession number GSE160763). The cell type was annotated according to our previous publication (PMID: 37269057). The genes included for ROS score evaluation were collected from HALLMARK_REACTIVE_OXYGEN_SPECIES_PATHWAY in the molecular signature database of GSEA (https://www.gsea-msigdb.org/gsea/index.jsp, Systematic name: M5938). Student’s t-tests were performed.

**Supplementary Figure 4**


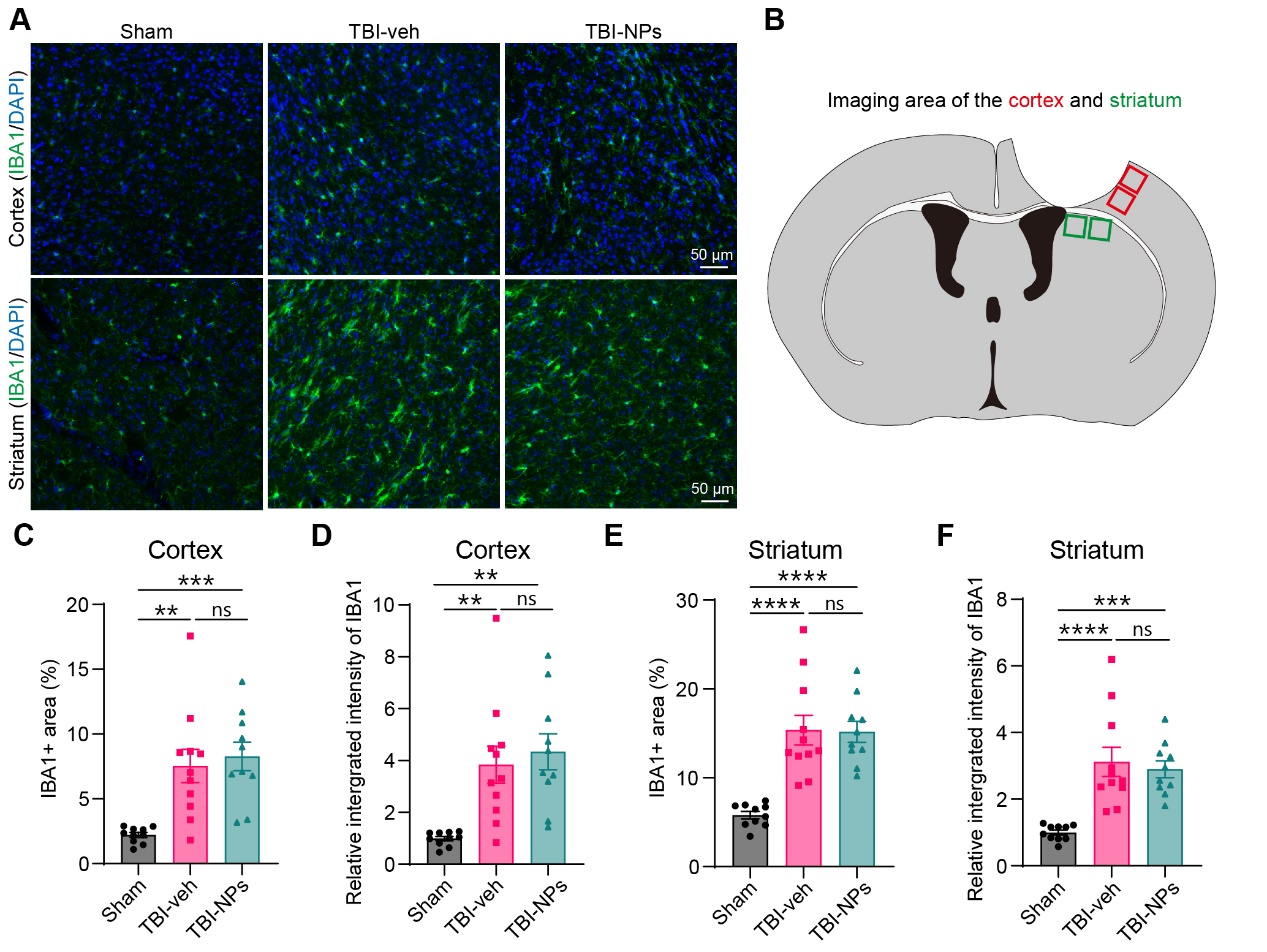
**Supplementary Figure 4.** Treatment with the Co_3_O_4_ NPs plays a limited role in IBA expression in microglia after TBI. (A) Representative images of IBA1 immunostaining in the cortex and striatum of mice with different treatments. Scale bar: 50 µm. (B) Schematic illustration of the image field for data analysis. (C-D) Quantification of IBA1-positive area (C) and the relative integrated intensity of IBA1-positive cells (D) in the cortex of mice with different treatments. (E-F) Quantification of IBA1-positive area (E) and the relative integrated intensity of IBA1-positive cells (F) in the striatum. One-way ANOVA followed by Tukey's HSD test. n=10 in sham group, n=11 in TBI-veh group, n=10 in TBI-NPs group. ***p*< 0.01, ****p*< 0.001, *****p*< 0.0001.

**Supplementary Figure 5**


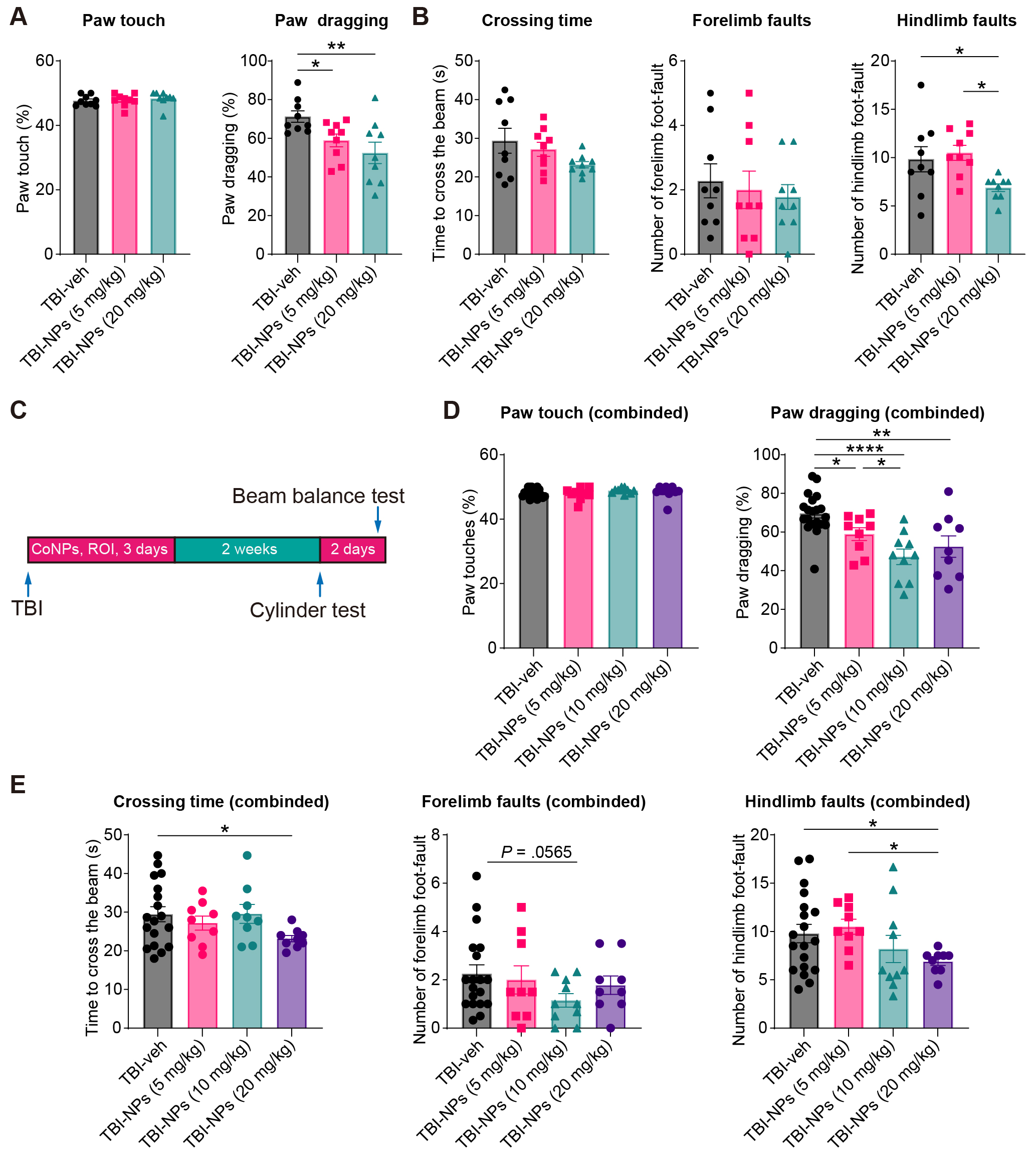


**Supplementary Figure 5.** Dose-dependent enhancement of neurological recovery by Co₃O₄ NPs treatment post-TBI. (A) Quantification of the percentage of paw touches and paw dragging in the cylinder test with low (5 mg/kg) and high dose (20 mg/kg) of Co₃O₄ NPs treatment. Both low (5 mg/kg) and high (20 mg/kg) doses of Co_3_O_4_ NPs significantly decreased paw dragging behavior in the cylinder test. (B) Quantification of the time to cross the beam and the number of forelimb and hindlimb foot-faults in the beam balance test. Treatment with the high dose (20 mg/kg) of Co₃O₄ NPs significantly reduced hindlimb foot-faults, whereas the low dose (5 mg/kg) had no effect. Sample size: n = 9 each group in A and B. (C) Flow chart of behavior tests. (D and E) Combined results of cylinder test (D) and beam balance test (E) with the different doses of Co₃O₄ NPs treatment post-TBI. Co₃O₄ NPs exert dose-dependent therapeutic effects in TBI-induced motor impairment. Original sample size in D and E: n=20 in the TBI-veh group, n=9 in the TBI-NPs (5 mg/kg) group, n=10 in the TBI-NPs (10 mg/kg) group, and n=9 in the TBI-NPs (20 mg/kg). Outliers were excluded using the ROUT method (Q=1%). One-way ANOVA followed by Fisher's LSD test. **p*< 0.05, ***p*< 0.01, ****p*< 0.001, *****p*< 0.0001.

**Supplementary Figure 6**


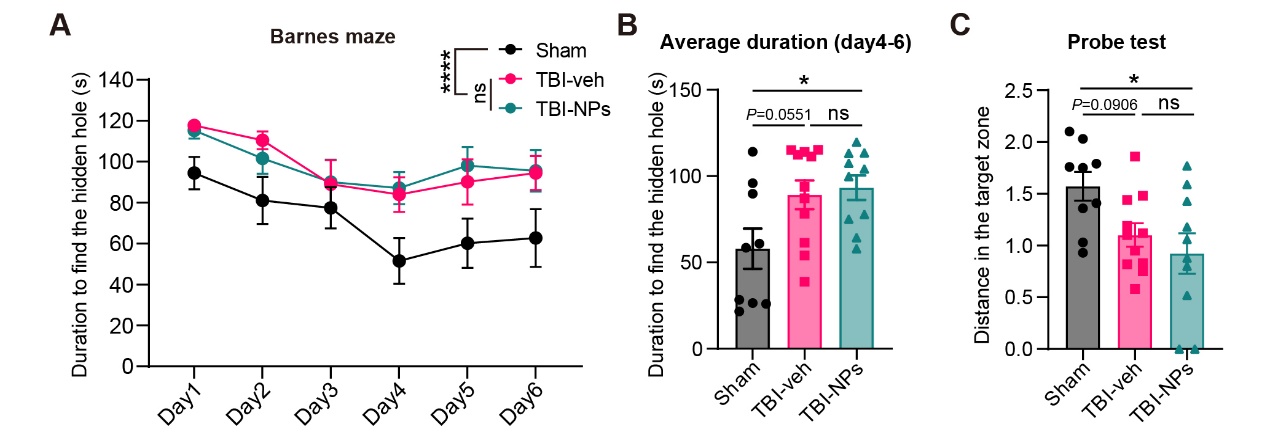


**Supplementary Figure 6.** The results of the Barnes-Maze test after treatment with Co_3_O_4_ NPs. (A) Data from the Barnes maze test over six days. (B) Bar graph showing the average duration of days 4-6 to find the hidden hole. (C) Bar graph showing the total distance moving in the target zone in the probe test of the Barnes maze test. Original sample size: n=10 in the sham group, n=11 in the TBI-veh group, and n=10 in the TBI-NPs group. Outliers were excluded using the ROUT method (Q=1%). Two-way ANOVA followed by Tukey's HSD test was performed to compare the main effect in A. One-way ANOVA followed by Tukey's HSD test in B and C. **p*< 0.05.

**Supplementary Figure 7**


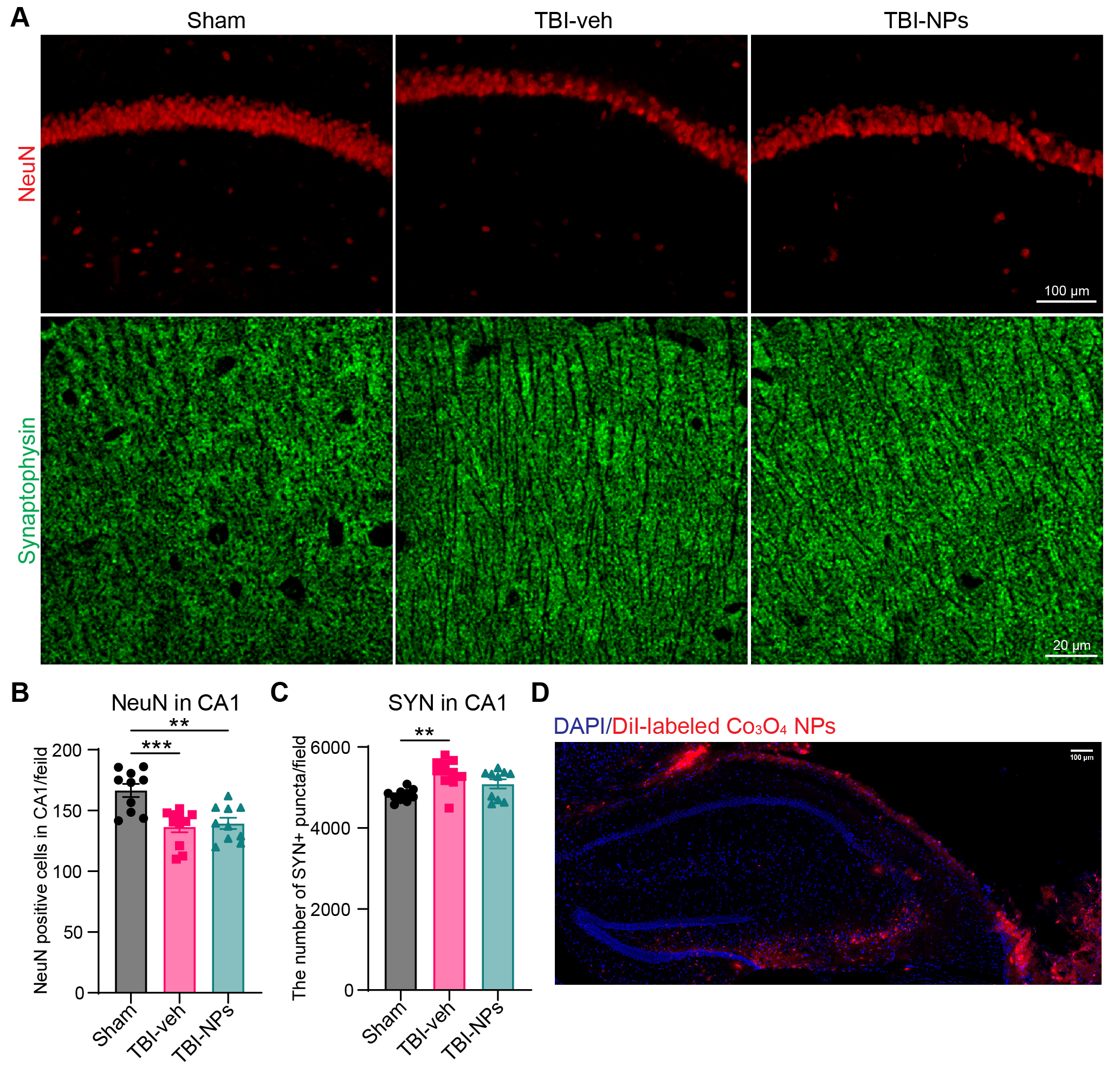


**Supplementary Figure 7.** Co₃O₄ NPs exhibit no effect on hippocampal CA1 neuron survival and aberrant synaptogenesis in the chronic phase of TBI. (A) Representative images showing hippocampal CA1 neuron (NeuN immunostaining) and synapses (synaptophysin, SYN immunostaining) in the chronic phase of TBI. Scale bar: 100 μm for NeuN images, 20 μm for SYN images. (B) Quantification of NeuN-positive neurons in CA1. (C) Quantification of SYN-positive puncta in CA1. (D) Distribution of DiI-labeled Co₃O₄ NPs in the hippocampus. DiI-labeled Co₃O₄ NPs were primarily deposited in the damaged brain region, and few were observed in the hippocampal CA1 region. Scale bar: 100 μm. One-way ANOVA followed by Tukey's HSD test. n=10 in the sham group, n=11 in the TBI-veh group, and n=10 in the TBI-NPs group. ***p*< 0.01, ****p*< 0.001.

**Supplementary Table 1**. Primary and secondary antibodies

| Antibody |  | Species | Distributor | Cat. # | Dilution |
| --- | --- | --- | --- | --- | --- |
| Primary antibodies | GFAP | Mouse | Sigma-Aldrich | G3893 | 1:1000 |
|  | GFAP | Rabbit | Proteintech | 16825-1-AP | 1:500 |
|  | IBA1 | Goat | Novus Biologicals | NB100-1028 | 1:600 |
|  | IBA1 | Rabbit | HUABIO | ET1705-78 | 1:500 |
|  | NeuN | Rabbit | Cell Signaling | 12943S | 1:600 |
|  | NOX2 | Rabbit | Proteintech | 19013-1-AP | 1:500 |
|  | 8-OHdG | Rabbit | Bioss | bs-1278R | 1:400 |
|  | CD68 | Rabbit | HUABIO | HA722285 | 1:500 |
|  | CoraLite® Plus 488-conjugated Synaptophysin antibody | Mouse | Proteintech | CL488-67864 | 1:1000 |
| Secondary antibodies | Anti-rabbit IgG, BF594 | Goat | Bioss | bs-0295G-BF594 | 1:1000 |
|  | Anti-rabbit IgG, DyLight 594 | Donkey | Boster | BA1147 | 1:1000 |
|  | Anti-rabbit IgG, BF488 | Goat | Bioss | bs-0295G-BF488 | 1:1000 |
|  | Anti-mouse IgG, BF488 | Goat | Bioss | bs-0296G-BF488 | 1:1000 |
|  | Anti-goat IgG, Alexa Fluor 647 | Donkey | Jackson ImmunoResearch | 705-605-003 | 1:1000 |
